# Comparison of Complications and Short Term Results of Conventional Technique Versus New Technique During Graft Ureteral Stent Insertion in Bari Technique at Emam Khomeini Hospital, Urmia

**DOI:** 10.5539/gjhs.v6n7p31

**Published:** 2014-09-18

**Authors:** A. Taghizadeh-Afshari, M. Alizadeh, S. Farshid

**Affiliations:** 1 Nephrology and Kidney Transplant Research Center, Urmia University of Medical Sciences, Urmia, Iran; 2 Urmia University of Medical Sciences, Urmia

**Keywords:** Kidney transplantation, Ureteroneocystostomy, Ureteral stents, Collection, UTI

## Abstract

**Introduction:**

There is debate on renal graft stenting during ureteroneocystostomy, patients with ureteral stents may encounter several complications such as encrustation, stent crustation which can lead to loss of kidney, and complications related to stent extraction: pain and UTI increasing related to cystoscopy for stent extraction accompanying excess expenses. This study designed to reduce complications related to stent extraction.

**Material and Methods:**

90 patients prepared for renal transplantation during 1 year randomly classified to groups, study group: patients with attached stent to Foley catheter, control group: patients with conventional technique (stent separated from Foley) then in their follow up; UTI, stent crustation, luts severity compared to each other.

**Results:**

Second week and fourth week UTI reported 25.6%, 2.3% in study group versus 34.9%, 4.7% in control group (P value 0.48 and 0.5). Urinary leakage was 3.3% overall, that all of them occurred in separate stent group, 37.5% vs. 0% in the linked stent group. Stent crustation in separate stent was 25% compared with 0% in the linked stent.

**Conclusion:**

Low complications rate in linked stent group, despite the lack of significant statistical differences, but indicate the effectiveness and success of the new technique.

## 1. Introduction

Double J. stents have different uses in urology in renal implantation is used for reducing ureteral leakage in preventing ureteral stenosis to bladder (Rajaian & Kumar, 2010; Mangus & Haag, 2004; Fayek et al., 2012a, 2012b) Many complications following a kidney transplant are due to poor technique of anastomosis or ureteral ischemia, although the routine use of stent in ureteral anastomosis to bladder in the extracorporeal urinary is routinely discussed (Rajaian & Kumar, 2010) but it has been observed that the use of stent is associated with reduced complications compared with those without a stent (Mangus & Haag, 2004; Fayek et al., 2012a). But using stent causes complications such as increasing infections and stoning stents (Rajaian & Kumar, 2010) and the difficulties imposed on the patient during removing the stent such as the use of anesthesia or general anesthesia to remove the stent that these problems causes tend to use the stent is reduced. There are different techniques to remove stent that include: with the help of fluoroscopy (Robertson, Edwards, & Harden, 1993) associated with radiation exposure to the patient’s body and cannot be used in some patients, the next with the help of the preceding flexible or rigid cystoscopy that due to low tolerance for pain, some patients require regional or general anesthesia, which is common in most centers to remove the stent (Evans & Ralph, 1991; Haluk Söylemez et al., 2011). Finally, non-aggressive technique in which with the help of Foley ureteral stent linked with Foley is removed. In this non-aggressive method ureteral stent is removed usually earlier and without aggressive methods and anesthesia or general anesthesia. In this method due to short time survival, stent is decreased in the probable location of complications and due to less impose of complications to the patient is expected to be an acceptable method (Morris Stiff, 1998; Jonathon Olsburgh, 2004; Christian, 2012; Haluk Söylemez et al., 2011). Given that kidney transplant center of Urmia is one of the active centers all over the country and in this study unlike other studies complications are compared separately it is expected in the project that is performed for the first time in the area effects and benefits of this approach are reviewed as short-term.

## 2. Methods

90 patients that are supposed to undergo renal transplantation were randomly divided into two groups. Techniques and processes similar in both groups after vascular anastomosis is usually renal vein to external iliac vein and renal artery to internal iliac artery. Ureteral anastomosis to bladder technique is Bari in both groups except that the first group during anastomosis of ureter to the bladder Double J stent was inserted that in the common technique one end of stent in the ureteral and the other end is inserted into the bladder, but in the second group new technique intra-end bladder of stent to the Foley catheter that is inserted into the bladder initially fixed with 0-4 vicryl suture then anastomosis is continued in both groups and the steps are the same. Patients in both groups are hospitalized one month in the section and in the first group stent are removed in the fourth week with cystoscopy but in the second group after a week stent are removed with the help of removing Foley. Patients are follow-up after discharge 1 to 2 times per week for 3 months, then every 1 to 2 weeks to 6 months, then every 4 to 6 weeks to 12 months. In this case, inpatient and outpatient patients with complications such as urinary leakage and urinary infection and stoning stent and difficult removing of stent and severity of urinary symptoms, patients are examined based on IPSS questionnaire. According to the similar studies, in this study that is conducted with RCT method, 90 patients with inclusion criteria were randomly assigned to two groups of control and study using a random numbers table. For this study, SPSS 16 software is used for data analysis. (Noruzy et al., 2013).

## 3. Results

A total of 90 patients were transplanted in this study, 4 patients were excluded due to the rejection of transplantation, 2 patients were in attached stent group, and 2 patients were in the separated stenting procedure. Finally, the study was performed with 86 transplanted patients in two control group (43 patients with separated stent and 43 patients with attached stent connected to catheter duct) and study group were enrolled. In the control group (separated stent procedure), 27 patients (62.8%) were male and 16 patients (37.2%) were female. In the study group (attached stent procedure) 26 patients (60.5%) were male and 17 patients (39.5%) were female. The mean age of the study group was 15.63 ± 44 and the control group was 15.20 ± 40.60 years.

## 4. Conclusions

Cause of transplant in the control group, 6 patients (14%) PCKD, 1 patient (2.3%) SLE, 15 patients (34.9%) HTN and 9 patients (20.9%), diabetes, 2 patients (4.7%), reflux, 5 patients (11.6%), kidney stones, 3 patients (7%), nephrotic syndrome and in 2 patients (4.7%) were associated with idiopathic etiology. Patients in the study group, 6 patients (14%) PCKD, 1 patient (2.3%) SLE, 26 patients (60.5%) HTN, 4 patients (9.3%), 2 patients (4.7%) reflux, 2 patients (4.7%) kidney stones, and in 2 patients (4.7%) were associated with idiopathic etiology.

**Number of transplants:** In the control group, 39 patients (90.7%) were transplanted for the first time and in 4 patients (9.3%) were transplanted for the second time. In the study group, 38 patients (88.4%) were transplanted for the first time and in 5 patients (11.6%) were transplanted for the second time.

**Frequency of second week urinary infection:** Prevalence of urinary tract infection in the control group during the second week, 15 patients (34.9%) were positive and 28 patients (65.1%) were negative and in the second week of the study group, the prevalence of urinary tract infection in 11 patients (25.6%) were positive and in 32 patients (74.4%) were negative. According to Chi-square test, there was no significant differences between the two groups in terms of infection in admission (P=0.48) (Table 1).

**Table 1:** Distribution of absolute and relative frequency of infection in the two groups

Stenting procedure	Infection	

	Positive	Negative
Separate	15(34.9%)	28(65.1%)
Attached	11(25.6%)	32(74.4%)
Total	26(30.2%)	60(69.8%)

Surveying infection during discharge time (one month after transplant) patients, results showed that of 43 patients in the control group, 2 patients (4.7%), urinary tract infection was positive and in 41 patients (95.3%) was negative and of 43 patients in the study group 1 patient (2.3%), urinary tract infection was positive and in 42 patients (97.7%) were negative. According to the Fisher Exact test there was no statistically significant differences between the two groups regarding urinary tract infection during discharge (P=0.5) (Table 2).

**Table 2:** Distribution of absolute and relative frequency of urinary tract infection one month after transplantation in both groups

Stenting procedure	Urinary tract infection one month after transplantation	

	Positive	Negative
Separate	2(4.7%)	41(95.3%)
Attached	1(2.3%)	42(97.7%)
Total	3(3.5%)	83(96.5%)

**Number of brain death frequency:** Of the 43 patients in the control group, transplantation in 2 patients (4.7%) were of brain death and in 41 patients (95.3%) were from living donors. Of the 43 patients in the study group, only 1 patient (2.3%) was transplanted from brain death and in 42 patients (97.7%) had received transplants from living donors. Urinary symptoms of patients during the first month was analyzed according to Luts results showed that: of 43 patients in the control group 27 patients (62.8%) were mild, in 12 patients (27.9%) were mod and in 4 patients (9.3%) were severe. Of the 43 patients in the study group, 30 patients (69.8%) AuA Mild and in 12 patients (27.9%) mod and in 1 patient (2.3%) were severe. According to Chi-square test there was no significant difference between the two groups of patients between Luts severity of patients. (P=0.37) (Table 3).

**Table 3:** Distribution of absolute and relative frequency of urinary symptoms in the first month according to Luts in the two groups

Stent	Urinary symptoms in the first month according to Luts		

	Mild	Mod	Severe
Separate	27(62.8%)	12(27.9%)	4(9.3%)
Attached	30(69.8%)	12(27.9%)	1(2.3%)
Total	57(66.3%)	24(27.9%)	5(5.8%)

The complications between the two groups, of 43 patients in the control group, 8 patients (19%) had complications after stenting and 37 patients (81%) had no complications after stenting.

Of the 43 patients in the study group, 4 patients (9.3%) had complications after stenting and 39 patients (90.7%) had not stent complications. Overall, 12 patients had complications and side effects and according to our findings is as follows.

**In the control group:** 3 cases urinary leakage (37.5%), 2 cases (25%) difficult removing of stent due to unusual positioning of the stent, ureteral obstruction in 2 cases (25%) due to forgetting to close the stent and stoning requiring reoperation and removing stent, 1 patient (12.5%) collection that was lymphoceles nature. (In 3 cases urinary leakage problem was solved with ureteroneocystostomy). 4 cases of complications in the attached group include lack of functioning of Foley and removing Foley earlier than a week was reported in 2 cases (25%), one case was due to the lack of functioning (25%) and one cases was due to the depletion of cuff liquid, 1 (25%) cases was due to distal ureteral stenosis caused by a blood clot and treated with expectant treatment, in 1 patient (25%) collection (peritoneum leakage) was reported. According to statistical analysis, Fisher Exact Test, there was no significant difference between the complications, which led to the departure of stent, and the two groups. (P=0.17) (Table 4). In the study group, 1 patient had ureteral obstruction problem that was fixed with nephrostomy.

**Table 4:** Absolute and relative frequency of complications (lack of Foley function) in the two groups

Complications (lack of Foley functioning	Stenting procedure						

Collection	Distal stenosis	Cuff liquid depletion	Lack of Foley functioning	Ureteral obstruction	Difficult removing	Ureteral leakage	
1(12.5%)	0(0%)	0(0%)	0(0%)	2(25%)	2(25%)	3(37.5%)	Separate
0(0%)	1(25%)	1(25%)	1(25%)	1(25%)	0(0%)	0(0%)	Attached
1(8.3%)	1(8.3%)	1(8.3%)	1(8.3%)	3(25%)	2(16.7%)	3(25%)	Total

According to the test results obtained by Kaplan-Meier, P. value showed that the follow-up period had no effect on removing stent. (P=0.64)

## 5. Discussion

Ureteral stent in urologic surgeries to reduce complications has long been considered. In recent years, despite differences in terms of using stent in transplant surgeries, the ureteral stent is mounted. Ureteral stents in renal transplantation center, Urmia routinely used in transplantation surgeries (Mohamadi et al., 2011). Given underlying conditions of transplant patients such as immunodeficiency or other underlying diseases long-term survival of stent in transplant patients can be associated with complications which can lead to transplanted kidney failure. However, removing the stent in patients with conventional methods (using cystoscopy) increased infection rates and costs and pain while removing stent that for reducing these complications in this study, we used G-Morris et al. (Evans & Ralph, 1991) that was conducted as Pilot in 1998. G-Morris study was aimed at reducing urinary sepsis. Urinary leakage is one of the most important complications in transplant patients, which can cause kidney failure. In a study of Hafez et al. (2011) a population of 101 people, the technique Lich-gregoir anastomotic was used and in all patients stent was used. Urinary leakage rate was 2%. In another study by M. EL-MEKRESH et al. (2001) conducted on 1,200 patients that various techniques of urethral anastomosis to bladder are used that the preferred technique was Lich-gregoir and without stenting leakage rate have been reported in 35 cases. Also in the same study (Sameh et al., 2012) Sameh A. urinary leakage rate in without stent group was 2.8% and in the stent group was 1.8% that in our study that this rate was reported in 3 cases (3.3%) that all 3 cases were in separated stent group, with the exception of ureteral anastomosis technique used in our study was Bari. And all cases of leakage, observed in the separated stent that two cases in the second week, and 1 case after the first month, referred with hydronephrosis. In the group that stenting was separated from ureter that these findings were in contrast with the results expected because despite the longer stent, this complication was due to attached stent group that due to independent factors such as ischemia of distal ureteral and technical errors. All 3 cases were improved by re-treatment, whereas we expected based on studies that did not use stents higher leakage was created that the difference could be related to our anastomosis technique. Forgotten stent is also one of the important complications that in our study 2 cases were in separated group that one case after 3 months and one case after 1.5 months was referred with stone stent that underwent TUL and stent was removed. The other 2 patients were because of the difficulty in removing the stent due to stent migration upward with local procedure underwent general anesthesia and by ureteroscopy stent was removed. But in the attached stent group, there was no problem in removing stent during removing Foley. In attached stent group there was one distal ureteral stricture case that improved after embedding nephrostomy and expect treatment. But a similar complication was not seen in the separated stent group, while in Tavakoli et al. (2007) study in non-stent group stenosis was 7.7% compared with 0% in the stent group. In this study time period of stent was 2 weeks that was lower than our study (4 weeks), which could be indicative of the difference.

As the transplant patients infected with immunodeficiency are exposed to more urinary infection. In Shrestha BM (Shrestha, 2006) study total rate of urinary infection in transplant patients was 43%, but statistically there was no significant difference between stent and non-stent groups. In Chordia et al. study, 212 patients that ureteral stent was used during anastomosis with 183 non-stent patients in terms of amount of bacteriuria at the first 6 weeks (stent was in ureter) and then 6-12 weeks that stent was removed and after 1 year was compared that the difference was not significant. In this study the long-term viability of stent was not a risk factor for UTI instead long-term urethral catheter, causing bacteriuria. In the study of W. Parapiboon (W. Parapiboon, 2012) the rate of bacteriuria in the first week was the highest amount which is more associated with longer survival (7 days) urethral catheter, but at the end of the first month infection rate have been reported 23%. In this study we compared ureteral infection in 2 times in second week (after removal of the catheter lumen) and the end of the first month in 2 group that 26 cases (33.2%) in the second week, and 3 cases (3.5%) one month after transplant urinary tract infection were positive. And separately in 5 cases (34.9%) out of 43 patients with urinary tract infection in separated stenting procedure and 11 cases (25.6%) out of 43 patients in the attaches stenting procedure, urinary tract infection were positive, one month after transplantation, 2 cases (4.7%) in the separated stenting procedure and 1 case (2.3%) in the attached stenting procedure were positive for UTI. In this study, for the removal of the effects of urethral catheter in bacteriuria, we compared urine culture in the second and fourth weeks (after catheter removal).

Symptoms of lower urinary tract in patients with urologic associated with the presence of the ureteral stent, perhaps due to defying the trigone removing stent with activity and stimulate the lining of the bladder (Rane et al., 2001; Sur et al., 2008). In the study by Ricardo et al. (2009) severity of luts stimulation in patients, who underwent stent for various reasons, had been investigated. Luts was in 80% of patients with a preferred frequency and urgency in 60-70% of cases. In this study severity of Luts depending on, mild, mod and severe compared between the two groups that in our study, there was no significant difference between the severity of luts in the group that stent was removed earlier with the group that stent was removed later (P = 0.37) (urinary symptoms in separate stenting procedure, 27 cases (62.8%) had mild, 12 cases (27.9%) mod and 4 cases (9.3%) severe and in the attached stenting procedure, 30 (69.8%) had mild, 12 cases (27.9%) mod and 1 cases (2.3%) severe. Since in the patients transplanted the new ureteral orifice is usually away from trigone thus the probability of stent and trigone contact is less also disability of patients’ associates can cause retentive symptoms of Luts. Cystoscopy for removing stent in transplanted patients compared with cystoscopy for removing stent in patients with ureteral orifice is more difficult in the natural place because in in patients with ureteral orifice in the ceiling and sometimes the tip of the stent is placed in the bladder neck usually causes difficulty in the procedure. Due to imposed pain on the patient, removing the stent is performed in some centers in the operating room under local anesthesia or general anesthesia. The average time required to remove stent in patients with orifice ureter at the normal place from preparing patients and cystoscopy equipment in women less than 1 min and in men 1.5 minutes and in patients transplanted in women 1.5 minutes and in men 1.5 to 2 minutes, which can indicate the difficulty of procedure in these patients. (This time was obtained by making a senior associate in 10 patients who were treated with endoscopic stone and stent transplant compared with patients who had been admitted to remove the stent), while the stents in patients who were linked to the Foley removing stent is possible with deflation Foley cuff that it also can be done by nurses. However, the cost of patients referred to an outpatient center to remove stent with insurance was averaged 8 dollars and uninsured 43 dollars. While the patients to remove the stent under anesthesia with insurance was 22 dollars and uninsured 172 dollars, while in patients with attached stent does not impose any additional costs to patients.

## 6. Design Limitations

- Small sample size to investigate the complications
- Lack of awareness of the severity of LUTS patients before stenting
- Failure to differentiate between men and women with severe LUTS
- Failure to analyze and compare patients in terms of satisfaction from cystoscopy under local anesthesia or general anesthesia compared with local procedure

## 7. Conclusion

Results of this study showed a low rate of complications such as infection and urinary leakage in patients with stent attached to the Foley compared with the separated stent is (although was not statistically significant) and the other costs imposed on patients and pain in patients during removing the stent is less that attached stent. These results indicate the usefulness and alternativeness of the conventional technique with this method, which required similar studies with larger sample sizes.
